# Sonic Anemometry to Measure Natural Ventilation in Greenhouses

**DOI:** 10.3390/s111009820

**Published:** 2011-10-19

**Authors:** Alejandro López, Diego Luis Valera, Francisco Molina-Aiz

**Affiliations:** Department of Rural Engineering, University of Almería, Ctra. Sacramento s/n, 04120 Almería, Spain; E-Mails: alexlopez@ual.es (A.L.); fmolina@ual.es (F.M.-A.)

**Keywords:** sonic anemometry, greenhouse, ventilation, insect-proof screens

## Abstract

The present work has developed a methodology for studying natural ventilation in Mediterranean greenhouses by means of sonic anemometry. In addition, specific calculation programmes have been designed to enable processing and analysis of the data recorded during the experiments. Sonic anemometry allows us to study the direction of the airflow at all the greenhouse vents. Knowing through which vents the air enters and leaves the greenhouse enables us to establish the airflow pattern of the greenhouse under natural ventilation conditions. In the greenhouse analysed in this work for *Poniente* wind (from the southwest), a roof vent designed to open towards the North (leeward) could allow a positive interaction between the wind and stack effects, improving the ventilation capacity of the greenhouse. The cooling effect produced by the mass of turbulent air oscillating between inside and outside the greenhouse at the side vents was limited to 2% (for high wind speed, *u_o_* ≥ 4 m s^−1^) reaching 36.3% when wind speed was lower (*u_o_* = 2 m s^−1^).

## 1. Introduction

Natural ventilation is the most common system used for greenhouse cooling and constitutes a key function in greenhouse climate control, being a major factor in their energy and mass balances, and hence in the control of crop growth and development [1,2]. Monitoring and control of the greenhouse environment play a decisive role in greenhouse production processes [3]. In order to optimise the design and operation of natural ventilation systems, one must first determine and understand, both qualitatively and quantitatively, the natural ventilation mechanisms.

The main driving forces of ventilation for a greenhouse equipped with both roof and side openings are caused by a combination of pressure differences induced by the following effects [4,5]: (1) the static wind effect due to the mean component of the wind velocity, which induces pressure differences (side wall effect) between the side and the roof openings [6] and pressure differences between the windward and the leeward parts of the greenhouse [1]; (2) the buoyancy forces (also called stack or chimney effect) generating a vertical distribution of pressures between the side and roof openings [7]; and (3) the turbulent effect of the wind, generated by pressure fluctuations of the wind velocity along and across the greenhouse openings [1,5].

The earliest studies on the circulation of air in greenhouses date back to the mid-20th century [8]. Since then, numerous researchers have shown interest in studying and understanding natural ventilation in greenhouses. Many different methods have been used for this purpose: scale models [9–11], tracer gas methods [2,4,12,13], CFD (Computational Fluid Dynamics) simulations [12,14–18] and direct *in situ* measurements using a variety of sensors [1,3,5,19–21].

Direct determination of the airflow through the openings of the greenhouse using sonic anemometry is the only experimental technique that allow the prediction of greenhouse air exchange rates as well as the characterisation of its components: a steady effect resulting from the combination of both mean wind-related and stack effects and a turbulent effect linked to wind speed fluctuations [1]. The relative importance of the roof and side opening areas is crucial for Mediterranean greenhouses which are characterised by a limited number of spans, small covered area, and continuous vents [4].

However, most of the studies using this technique were carried out for greenhouses equipped with only roof openings [1,20,21] or side vents [22] and there has been a lack of experimental data of ventilation airflow obtained in greenhouses equipped with both roof and side screened openings [23]. Thus, the objectives of this study were to determine the ventilation rate, air velocity and turbulence characteristics in a three-span greenhouse with continuous screened side and roof vents under natural ventilation conditions. The present work differs from previous studies in that it uses sonic anemometry to determine the flow patterns in a greenhouse with both roof and side openings and analyses the contribution of turbulent flow to greenhouse cooling.

## 2. Experimental Setup

### 2.1. Site and Greenhouse Description

**Figure 1.**
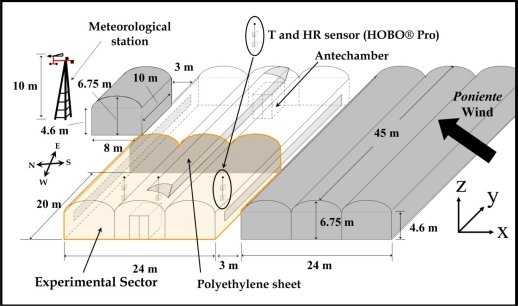
Location of the experimental greenhouse at the farm.

The experimental work took place in the 24 × 20 m^2^ western half of a 1,080 m^2^ three-span greenhouse located at the agricultural research farm of the University of Almería (36°51′N, 2°16′W). The experimental greenhouse was physically divided into two similar sectors by a polyethylene sheet fixed to a stainless steel structure, as this allows us to study the natural ventilation of each half separately for other research projects (Figure 1).

During the experiments both the side (1.05 × 17.5 m^2^) and roof vents (0.97 × 17.5 m^2^) were opened, giving an overall ventilation surface *S_V_* that represents 11.2% of the total greenhouse surface area *S_A_* (*S_V_/S_A_* = 0.112).

In order to prevent insects from entering the greenhouse, insect-proof screens were placed on all vents. The characteristics of the screens are as follows: 13 × 30 threads cm^−2^ (0.39 porosity; 164.6 μm pore width; 593.3 μm pore height; 165.5 μm thread diameter). The greenhouse contained a tomato crop (*Solanum lycopersicum* L. *cv.* Salomee) with an average height of approximately 1.38 m and a leaf area index (m^2^ leaf per m^2^ ground) of about 0.89.

### 2.2. Experimental and Instrumentation

Outside climatic conditions were recorded (frequency 0.5 Hz) by a nearby meteorological station placed at a height of 10 m (Figure 1). The meteorological station included a BUTRON II measurement box (Hortimax S.L., Almería, Spain) equipped with a Pt1000 temperature sensor and a capacitive humidity sensor (temperature measurement range −25 °C to 75 °C; accuracy ±0.01 °C; humidity range 0% to 100%; accuracy ±3%). Outside wind speed was measured with a Meteostation II (Hortimax S.L.), incorporating a cup anemometer (measurement range 0 to 40 m s^−1^; accuracy ±5%). Wind direction was measured with a vane (accuracy ±5°). Solar radiation was measured using a Kipp Solari (Hortimax S.L.) sensor (measurement range 0 to 2,000 W m^−2^; accuracy ±20 W m^−2^).

Temperature and humidity inside the greenhouse were measured (frequency 0.5 Hz) using six autonomous dataloggers (HOBO Pro Temp-HR U23-001, Onset Computer Corp., Bourne, MA, USA), protected against direct solar radiation with a passive solar radiation open shield. The dataloggers were placed in a vertical profile under the ridge of the three greenhouse spans at heights of 1 and 2 m (Figure 1). These fixed devices measure a temperature range of −40 °C to 70 °C (accuracy ±0.18 °C) and relative humidity range of 0% to 100% (accuracy of ±2.5%).

Measurement tests were carried out under prevailing *Poniente* southwest (SW) wind, one of the most common in the province of Almería. The outside climatic conditions remained relatively stable for the duraction of the ten measurement tests (Table 1).

**Figure 2.**
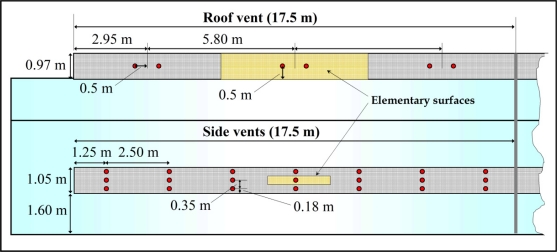
Measurement points at the lateral vents and at roof vents.

**Figure 3.**
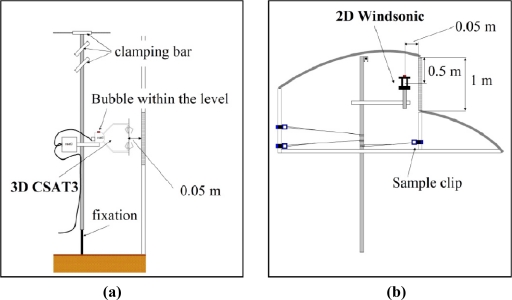
Details of the experimental setup using 3D anemometers placed at the side vents (**a**) and 2D anemometers at the roof vent (**b**).

The three components of air velocity and temperature at the side vents were measured with two 3D sonic anemometers (mod. CSAT3, Campbell Scientific Spain S.L., Barcelona, Spain; accuracy ±0.04 m s^−1^ and ±0.026 °C). Each side vent was divided into seven equal vertical spaces and three horizontal ones (Figure 2). Air velocity was measured at the centre of each of the resulting 21 spaces, moving the trisonic anemometers from one point to another [Figure 3(a)]. At each point the sonic anemometer measured at a sampling rate of 10 Hz for 3 min.

The mean surface corresponding to each measurement point at side vents was 0.9 m^2^. This value is less than those used to calculate the ventilation flux by other authors: through the only roof vent of a tunnel greenhouse (2.6 m^2^ per point) [19], in a mono-span greenhouse with two side vent openings (1.1 m^2^ per point) [22], in a four-span greenhouse with three roof vents (8.5 m^2^ per point) [20] and in a five-span Almería-type greenhouse (2.1 m^2^ per point) [23]. The mean surface corresponding to each equal surface in the roof vent is 5.7 m^2^.

As it proved difficult to displace the anemometers along the roof vent, this vent was divided into three equal surfaces and the air velocity measurements were taken continuously at the centre of each surface (Figure 2) by six 2D sonic anemometers (mod. Windsonic, Gill Instrument LTD, Lymington, Hampshire, UK; accuracy 2%) fixed to the greenhouse structure [Figure 3(b)]. Data from all sonic anemometers were recorded by two CR3000 Microloggers (Campbell Scientific Spain S.L.), with a data registration frequency of 10 Hz [21] and 1 Hz, respectively, for the 3D and 2D sonic anemometers. One 3D and three 2D sonic anemometers were connected to each micrologger.

Two 3D sonic anemometers were used to determine the air velocity at the side vents [Figure 3(a)], one per vent, and taking measurements at each point over 3 min. This time period is a compromise between a shorter one that may reduce accuracy and a longer one that may increase the overall difference with regard to outside microclimate parameters [23]. Six 2D sonic anemometers were used to measure the air velocity at the roof vent [Figure 3(b)], taking continuous measurements.

### 2.3. Analyses

Ultrasonic anemometers are able to determine the air velocity vector and the sonic temperature. They are instruments suitable, widely used devices to evaluate turbulent parameters such as mean air velocity, turbulence intensity and integral length scale. In this work we have calculated and studied the different parameters described below.

#### 2.3.1. Mean and Turbulent Air Velocity

For air velocity *u* [m s^−1^] and its components (*u_x_*, *u_y_* and *u_z_*; Figure 1), the mean air velocity measured over a period *Δt* is [24]:

(1) $$\bar{u}=\frac{1}{\Delta t}\int_{t}^{t+\Delta t}u\;\mathrm{dt}$$

We have also calculated the average value of the two-dimensional resultant of air velocity in the XY plane (*l*) and in the XZ plane (*v*), *u*(*t*) is the instantaneous air velocity, which can be expressed as the sum of time-mean value u and a fluctuating component *u′*(*t*) [24]:

(2) $$u(t)=\bar{u}+u\prime (t)$$

The variance of an air velocity over a period of time *Δt* is defined as [24]:

(3) $$\sigma^{2}=\bar{{u\prime }^{2}}=\frac{1}{\Delta t}\int_{t}^{t+\Delta t}{\left(u-\bar{u}\right)}^{2}\mathrm{dt}$$

Turbulence intensity *i* is standard deviation *σ* divided by mean local velocity *u*, so [24]:

(4) $$i=\frac{\sqrt{\bar{{u\prime }^{2}}}}{\bar{u}}=\frac{\sigma }{\bar{u}}$$

#### 2.3.2. Turbulence Macroscale

The normalized autocorrelation function *R*(*t*) is the correlation between air velocities at a fixed position at two different instants, *t* and *t* + *δ_t_* [25,26]:

(5) $$R(t)=\frac{\bar{u\prime \left(t\right)\cdot u\prime \left(t+\delta_{t}\right)}}{\sigma^{2}}$$

As opposed to integrating *R*(*t*) to infinity, it can only be integrated to the first zero crossing (*t_0_*) to obtain *t_int_*, the integral time scale [25]:

(6) $$t_{\text{int}}=\int_{0}^{t_{0}}R(t)\cdot \mathrm{dt}$$

and *L_i_*, the integral length scale [m], also called the macroscale [27] or the average size of the largest eddies [28]:

(7) $$L_{i}=\bar{u}\cdot t_{\text{int}}$$

#### 2.3.3. Discrete Energy Spectrum

According to turbulence theory, turbulent flow can be regarded as the superposition of eddies of different scales [29]. The spectrum of energy density, *E*(*f*) [m^2^ s^−1^], gives the relationship between the frequency of a signal *f* and the energy of the corresponding eddies. The discrete energy spectrum *E*(*f*) is calculated by [29]:

(8) $$E\left(f\right)=2\Delta t\;N^{-1}{\left|X\left(f\right)\right|}^{2}=2\Delta t\;N^{-1}X\left(f\right)\cdot X*\left(f\right)$$

where *X*(*f*) is the Fast Fourier Transform (FFT) of sample data *X*(*t*) of instantaneous velocity, and X*(*f*) is the conjugate complex number of *X*(*f*).

The turbulent flow consists of a mass of eddies of different scales. The average negative slope (*β* value) of the logarithmic power spectrum curves is the main parameter used in the analysis of airflow. The *β* value can reflect the energy distribution of eddies of different scales. Its relationship with *E*(*f*) can be expressed as [24]:

(9) $$E\left(f\right)\propto f^{-\beta }$$

A mechanically generated airflow is characterized by energy density spectra of low slope [29]. The slope of the energy spectrum for airflows generated naturally at the ventilation surfaces usually corresponds to an isotropic distribution of turbulence, *β* = 5/3 [30].

Total turbulence kinetic energy *k* [m^2^ s^−2^] can be calculated by the following expression [25]:

(10) $$k=\frac{1}{2}\left(\sigma_{x}^{2}+\sigma_{y}^{2}+\sigma_{z}^{2}\right)$$

where *σ_x_*, *σ_y_*, and *σ_z_* are the standard deviations of the three air velocity components. The turbulence energy dissipation rate *ɛ* [m^2^ s^−3^] is defined as [25]:

(11) $$ɛ=k^{3/2}\lambda^{-1}$$

#### 2.3.4. Anemometric Measurement of Volumetric Flow Rate

The mean and turbulent volumetric flow rates through the greenhouse were calculated by multiplying the scaled time-mean component *u_j_* and fluctuating component *u*′, respectively, of the air velocity perpendicular to the plane of the opening to the elementary surface *S_Vj_* in order to describe the air circulation through the opening [19]:

(12) $${\bar{G}}_{j}=\sum_{j=1}^{n}S_{Vj}\;{\bar{u}}_{j}$$

(13) $$G_{j}^{\prime }=\sum_{j=1}^{n}S_{Vj}u_{j}^{\prime }$$

With only two possible sampling positions at any one time in lateral vents, a difficulty arises from how to deal with changing external conditions over the time needed to take measurements at the different positions in the lateral vents of each sector (Figure 2). This problem can be overcome by selecting measurements for a fixed external wind direction and correcting the air velocities measured by the 3D sonic anemometers at each position *j* at the lateral vents *u_j_*(*t*) by scaling with the wind speed [23]. We have calculated a scaled air velocity in the opening, u^*^_j_(*t*), taking only wind effect into account, multiplying measured values of air velocity *u_j_*(*t*) at minute *t* at each point *j* in the greenhouse openings by the ratio between the average wind speed for the overall test period (several hours) and the instantaneous values *u_o_*(*t*) (average for each minute *t*):

(14) $$u_{j}^{*}\;\left(t\right)=u_{j}\;\left(t\right)\frac{{\bar{u}}_{o}}{u_{o}\left(t\right)}$$

The tests took 2–3 hours on average, generating a considerable amount of data. Two programmes were designed to process the air velocity data using MATLAB 7.0 [31], one for data obtained by the 3D sonic anemometers (frequency 10 Hz) and the other for data from the 2D sonic anemometers (frequency 1 Hz). These programmes allow us to analyse a complete experiment in a matter of a few minutes, while the first attempts at analysis using spreadsheets required hours of work.

#### 2.3.5. Estimation of the Sensible Energy Flux by the Eddy Correlation Method

Local estimations of mean and turbulent flux of sensible energy across a vent opening can be obtained using eddy correlation techniques [26]. Considering the transport of heat with a velocity *u* normal to the vent opening, then an elemental volume of air will transport heat at a rate *ρC_p_uT* across unit surface of the opening. Now using Reynolds formulation we may write the classic formula:

(15) $${\rho C}_{p}\bar{\mathrm{uT}}={\rho C}_{p}\bar{u}\bar{T}+{\rho C}_{p}\bar{u\prime T^{\prime }}$$

Thus, the flux is composed of a mean normal flow of air (*ρC_p_ūT̄*) and a part due to eddying motion ( ${\rho C}_{p}\bar{u\prime T^{\prime }}$) proportional to the covariance between *T* and *u* denoted by $\bar{u\prime T^{\prime }}$. Using this approach with 3D sonic anemometers, the mean and turbulent heat fluxes can be deduced [Equation (15)].

The sensible energy flux *Q* exchanged between the greenhouse air and outside can be expressed by different relations as follows. Assuming the air in the greenhouse mixes well, the sensible energy flux *Q* can by given as a function of the air exchange rate [1,5]:

(16) $$Q={\rho C}_{p}G\Delta T_{\mathrm{io}}={\rho C}_{p}\frac{S_{v}}{2}E_{Q}\bar{u_{o}}\bar{\Delta T_{\mathrm{io}}}$$

where *E_Q_* is the wind-related ventilation efficiency coefficient deduced from heat flux measurement.

Using the normalized form described by Boulard *et al.* [1] and calculating the air circulation through the opening for each elementary surface *S_Vj_* we can calculate the flux of sensible energy as:

(17) $$Q={\rho C}_{p}\sum_{j=1}^{n}\left[\left(\frac{{\bar{u}}_{j}\bar{{\left(T-T_{o}\right)}_{j}}}{u_{o}\Delta T_{\mathrm{io}}}+\frac{\bar{u_{j}\prime T_{j}\prime }}{u_{o}\Delta T_{\mathrm{io}}}\right)\bar{u_{o}}\bar{\Delta T_{\mathrm{io}}}S_{Vj}\right]$$

From direct measurements at the vent opening we can then obtain a mean value of the total sensible heat exchange in its normalized form as [1]:

(18) $$Q={\rho C}_{p}S_{V}\bar{u_{o}}\bar{\Delta T_{\mathrm{io}}}\left[\frac{\bar{u}\bar{\left(T-T_{o}\right)}}{u_{o}\Delta T_{\mathrm{io}}}+\frac{\bar{u\prime T\prime }}{u_{o}\Delta T_{\mathrm{io}}}\right]$$

## 3. Results and Discussion

The methodology followed permits in-depth study of the airflow characteristics at the greenhouse vents in order to determine the ventilation airflow pattern, to estimate the air exchange rate for each experiment and to analyse the turbulence characteristics.

### 3.1. Airflow Characteristics

**Figure 4.**
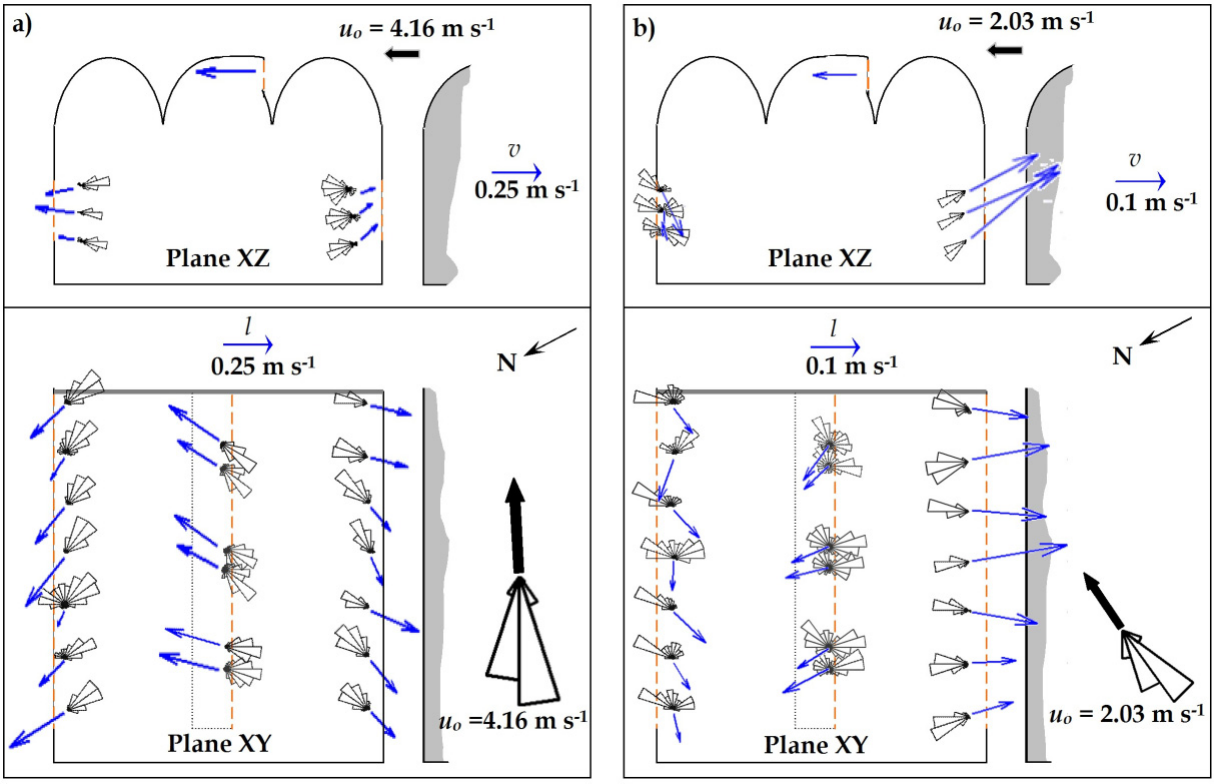
Polar histograms of the horizontal angle and projection of the air velocity at the middle of the side vents and at the roof vent. Experiments 2 **(a)** and 4 **(b)** carried out on 08/04/2009 and 07/05/2009, respectively.

Given the particular location of the greenhouse and the placing of the vents, when natural ventilation occurs under prevailing southwesterly *Poniente* winds, the wind and thermal effects are contrary. The wind causes air to enter through the windward roof vent and to leave through the leeward side vent (the windward side vent is obstructed by another greenhouse). The thermal effect, on the other hand, causes warm air to rise and leave through the roof vent, favouring the entrance of air through the side vents. The polar histograms in Figure 4(b) illustrate a similar distribution of air at the roof vent in the leeward and windward direction. This illustrate the entrance and exit of air through the roof vent, providing evidence of the negative interaction of the wind and thermal effects, although the wind effect predominates, as the direction of the mean vector of velocity indicates [Figure 4(b)].

The ventilation rate of the greenhouse is affected by the buoyancy effect generated by the difference in temperature between the inside and outside air Δ*T_io_*. Several authors have pointed out that the buoyancy effect is particularly relevant for winds of less than 1.8 m s^−1^ [13], 1.5 m s^−1^ [5] or 1 m s^−1^ [11]. When a vertical side wall opening was added to the roof window, the temperature effect was enhanced by the so called chimney effect, linked to with the vertical distance between the two openings, in such a way that it becomes negligible only for an external wind speed of over 4 m s^−1^ [32]. For greenhouses with both side and roof vents the thermal effect is considered to have a major bearing when the ratio *u_o_*/Δ*T_io_^0.5^* is less than 1 [4] or less than 0.3 [33].

In experiments 1, 2, and 3, when *u_o_*/Δ*T_io_^0.5^* was 1.5 or greater, very little air was seen to exit through the roof vent, which is a clear indication of the predominance of the wind effect over the thermal one, with air leaving through the side vents quite consistently [Figure 4(a)].

The wind can either assist the buoyancy force or oppose the airflow [34]. For opposing winds, we can observe the alternation of positive and negative flows (entrance and exit of air, respectively) at the roof vent [Figure 4(b)]. A certain discrepancy between wind direction and the direction of airflow entering the greenhouse through the roof vent was observed in some experiments. These differences may be due to the location of the meteorological station, which recorded the characteristics of the wind once it had passed through the experimental greenhouse.

In experiment 4, with a *u_o_*/Δ*T_io_^0.5^* ratio of under 1, the combination of opposing wind and thermal effects led to less uniform airflow at the vents, with alternating positive (entrance) and negative (exit) airflow at the roof vent [Figure 4(b)].

The use of sonic anemometry has allowed us to establish the ventilation pattern under different wind conditions. Under moderate-strong *Poniente* winds (*u_o_* ≥ 4 m s^−1^), the greenhouse is mainly ventilated by the wind effect, with air entering through the roof vent and leaving through both side vents. The combination of thermal and wind effects can lead to air leaving the greenhouse through the roof vent and/or entering through the side ones, although these flows which run contrary to the overall pattern occur infrequently.

Under weak *Poniente* winds (*u_o_* < 4 m s^−1^), there is a clear interaction between the thermal and wind effects at all three vents: at the roof vent air enters due to the wind effect and exits due to the stack effect, while at both the windward and leeward side vents air was also seen to enter and exit, entering in the main at the leeward side and leaving through the windward one. The airflow pattern observed is conditioned by the fact that on the windward side the greenhouse gives onto another greenhouse, without which the ventilation pattern would be completely different.

Under prevailing *Poniente* (SW) wind, in the experimental greenhouse a roof vent designed to open leeward (towards the North) allowed a positive interaction between the wind and stack effects, improving the ventilation capacity of the greenhouse. This result confirms that in certain circumstances (namely the obstructed windward side vent) a leeward roof vent can improve ventilation. Using CFD simulations Molina-Aiz [35] predicted a greater ventilation flux in an Almería-type greenhouse with two side openings, if the roof vents were opened towards the leeward side. Using the same method, Lee and Short [36] also observed better ventilation when the four roof vents opened towards the leeward side in a four and one-half span commercial greenhouse.

### 3.2. Evaluation of the Mean and Turbulent Ventilation Flows

By applying Equations (12–14), the volumetric flow rate at the three vents has been determined (leeward side vent *G_LS_*, windward side *G_WS_* and roof vent *G_WR_*), calculating the mean ventilation rate for the greenhouse *G_M_*. We have also calculated the air exchange rate from the greenhouse volume (2681.7 m^3^) and the mean flow (Table 2).

The accuracy of the mean values of air exchange measured can be assessed by totalling the entrance and exit flows measured at all vents. To verify to what extent the Law of Mass Conservation is met in the greenhouse [1,37], the error in the calculation of the ventilation flows has been estimated as follows:

(19) $$E_{G}=\frac{\Delta \bar{G}}{{\bar{G}}_{M}}=\frac{G_{\mathrm{LS}}\left(u_{x}^{*}\right)+G_{\mathrm{WS}}\left(u_{x}^{*}\right)+G_{\mathrm{WR}}\left(u_{x}\right)}{G_{M}}\times 100$$

The mean error in calculation of the airflows, *E_G_*, obtained was 16.3%. Following a similar methodology in an Almería-type greenhouse with two side and two roof vents the values of *E_G_* were between 3.0% and 37.0% [23]. Other researchers obtained errors of 2.2% and 2.6% [1] and 31.6% [37] in a multi-span greenhouse with only one roof vent.

The accuracy of this method of calculation of airflow depends mainly on the stability of the wind conditions (intensity and direction) and the influence of the thermal effect on greenhouse ventilation. Variable wind conditions or ones in which the wind or thermal effect does not clearly predominate may lead to less accuracy. The maximum error in calculating volumetric flow rate in these experiments occurred when the *u_o_*/Δ*T_io_^0.5^* ratio was close to 1.5. At such a ratio, the interaction between the wind and thermal effects produces instability in the direction of the airflow in the openings and can impair the accuracy of the velocity measurements recorded with the anemometers.

### 3.3. Evaluation of the Mean and Turbulent Sensible Energy Flows through the Side Vents

The cooling effect produced by the mass of turbulent air oscillating between inside and outside the greenhouse at the side vent is limited to 2% for the three first experiments (Table 3). However turbulence produces about 60% of the total airflow through the greenhouse opening (Table 2). This indicates that only a small proportion of the fluctuating airflow at the side vents mixed well with the bulk of air in the greenhouse. Usually, at the side of the greenhouse turbulence causes warm air to exit through the openings and mix with the cooler outside air. However, as the mean airflow heats the side zone of the greenhouse, the air that comes out due to turbulence has a much higher temperature than in inside the greenhouse. The heat loss through the side openings due to turbulence is therefore small.

Molina-Aiz [35] also measured the sensible heat flux through the vent openings (two side openings and two roof vents) of an Almería-type greenhouse. The turbulent volumetric airflow accounted for between 31% and 45% of the overall flow, whereas the turbulent flux contributed 3–14% of the total sensible heat transfer.

In the last experiment carried out, the cooling produced by the turbulent airflow accounted for 36.3% of the overall cooling effect at the side vents (Table 3). In this case, when wind speed was lower (*u_o_* = 2.03 m s^−1^), air entered the greenhouse through the leeward side vent [Figure 4(b)], which does not therefore contribute to the cooling of the greenhouse (Table 3). Furthermore, the air leaving the greenhouse due to turbulence has a much lower temperature than when inside of the greenhouse, increasing the covariance $\bar{u\prime T^{\prime }}$ and the sensible heat transfer due to eddying motion ( ${\rho C}_{p}\bar{u\prime T^{\prime }}$). The contribution of the turbulent flux measured in this case is similar to that reported in the roof vent of a bi-span greenhouse in France: 42% [26] and 23% and 45% [1].

The total sensible heat exchange in its non-dimensional form varied from 0.017 to 0.051 (Table 3), which is similar to the values of between 0.015 and 0.028 reported in an Almería-type greenhouse, equipped with insect-proof screens [35], and lower than those estimated in a bi-span greenhouse without nets: 0.06 [26] and 0.10 and 0.13 [1].

### 3.4. Turbulence Flow Characteristics

#### 3.4.1. Turbulence Intensity Levels

The turbulence intensity *i* at the windward side opening was similar for the four experiments, possibly due to the effect of the greenhouse close to this opening, which may impede the airflow through it. The increase in turbulence intensity caused by the outside obstacles was also observed in an Almería-type greenhouse with two side and two roof vents [23] blocked by a publicity sign 1.6 m away from a side vent.

The average levels of turbulence intensity observed at the vents (Table 4) are in some cases higher than those observed inside a mesh-covered greenhouse (83% porosity) in which *i* varied from 0.2–0.8 for *u_o_* < 0.5 m s^−1^ [38]. The turbulence intensity at the vents of a multi-span greenhouse with two side vents totally free of obstacles was greater at the windward vent than at the leeward one [22].

#### 3.4.2. Energy Levels and Measures of Turbulence Scales

The mean values of turbulence kinetic energy *k* (Table 4) are greater at the roof vent (windward), where the wind has a direct impact, transmitting much of its energy to the airflow entering the greenhouse. In a tunnel greenhouse the turbulence kinetic energy was also observed to be greater on the windward than on the leeward side [26]. The presence of an obstacle at the windward side vent brings about a drastic reduction in the energy dissipated by the airflow, and as a result in its capacity to mix and transport heat and water vapour [39].

The macroscale represents the dimension of the most energetic eddies that have a significant effect on the mixture of air and therefore on ventilation [40]. The importance of studying the macroscale lies in the fact that it may be related to the geometry of the vents [40] and to the location of the vents with respect to the wind and to adjacent buildings. This parameter is slightly lower for the blocked windward side vent (*L_i_* = 0.44 ± 0.28 m) and the roof vent (*L_i_* = 0.44 ± 0.40 m) than for the leeward side vents free of obstacles (*L_i_* = 0.59 ± 0.40 m). These values are similar to those measured in the openings of an Almería-type greenhouse with insect-proof screens, between *L_i_* = 0.25 m and 0.99 m [35].

The tight-mesh insect-proof screens reduced the turbulence level and increased the spectral decay rate, which implies the generation of smaller scales of flow, since small turbulent eddies are less energetic and the dissipation of energy in these scales is faster than in large ones [41]. Small eddies also have a much lower capacity to transport heat and water vapour out of the greenhouse than large-scale ones [41]. An important reduction of the macroscale was observed in our experimental greenhouse in the simultaneous measurement of air velocity with the 3D anemometers at several points outside and inside the greenhouse at the same distance from the screen [31].

#### 3.4.3. Discrete Energy Spectrum

**Figure 5.**
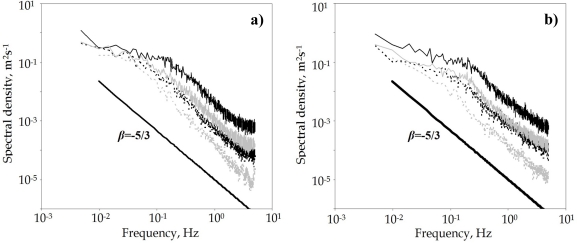
Energy density spectra for the longitudinal component, *u_x_*, at the northern **(a)** and southern **(b)** side vents. Experiments 1 ( 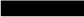), 2 ( 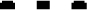), 3 ( 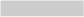) and 4 ( 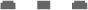).

Breaking down the time series into components of frequency allows us to observe how eddies of different scales contribute to overall turbulence (Figure 5). The energy density spectra shown in the figures of this section have been obtained by calculating the average spectrum from all those obtained at the 21 points at the side vents.

The level of the spectrum allows us to determine under which conditions the airflow at the side vents is most turbulent and energetic: the spectrum was higher when the wind speed was greater. The most energetic eddies are those which have the most significant effect on the mixture of air and therefore on ventilation [40].

The different values of the slope of the spectrum *β_x_* for the component perpendicular to the ventilation surface (Table 5) may be due to several reasons: the geometry of the vent [40,42], the use of insect-proof screens [39] or the influence of buoyancy [43].

In experiments 1, 2 and 3, when the wind speed was moderate to high and the thermal effect ran slightly contrary to the main direction of airflow generated by the wind effect, the air left the greenhouse through the leeward side vent and through the windward one (with an obstacle). In both cases the value of the slope of the spectrum was *β_x_* < 5/3. In experiment 4, when the wind speed was low and the thermal effect had a greater influence on airflow, the air entered and exited simultaneously (observing a high fluctuation in airflow direction) through the leeward side vent, *β_x_* >> 5/3.

## 4. Conclusions

Using sonic anemometry techniques it has proved possible to identify the vents through which air enters and exits the greenhouse, and therefore to establish natural ventilation flow patterns. Opening the roof vent to the windward side causes a combination of contrary wind and thermal effects in natural ventilation of greenhouses. As outside air enters it circulates downwards, contrary to natural convection due to the thermal effect.

Under certain circumstances, such as an obstructed windward side vent, a leeward roof vent can improve ventilation, allowing a positive interaction between the wind and stack effects. The contribution of turbulence to the overall sensible heat transfer between inside and outside the greenhouse through the side vents increased from 2% to 36.3% when the wind speed decreased from 4 to 2 m s^−1^.

The methodology presented in this work has allowed us to quantify accurately the greenhouse ventilation rate under varying conditions of outside wind. It has also allowed us to characterise the turbulence of the airflow at the vents, providing useful information for future validations of simulations based on CFD. The programmes designed in MATLAB 7.0 allow a 69% reduction in the memory capacity required to store the data generated during analysis.

## Figures and Tables

**Table 1.** Outside climatic conditions for the measurement tests. Average wind speed *u_o_* [m s^−1^], wind direction *θ* [°], outside and inside temperature *T_o_* and *T_i_* [°C], outside and inside humidity *HR_o_* and *HR_i_* [%], outside radiation *R_g_* [W m^−2^] and ratio determining the relative importance of the wind and thermal buoyancy forces *u_o_*/Δ*T_io_^0.5^*.

**Number-Date**	**Time**	***u_o_***	***θ*^a^**	***HR_o_***	***HR_i_***	***T_o_***	***T_i_***	***R_g_***	***u_o_*/Δ*T_io_^0.5^***
1-07/04/2009	11:52–14:47	6.86 ± 1.41	300 ± 7	67 ± 2	52 ± 7	17.5 ± 0.4	22.8 ± 1.9	527 ± 259	2.98
2-08/04/2209	10:49–13:31	4.16 ± 1.06	295 ± 15	29 ± 8	38 ± 2	18.3 ± 0.4	25.7 ± 1.4	562 ± 237	1.53
3-14/04/2009	11:21–14:01	4.01 ± 1.13	294 ± 10	72 ± 2	57 ± 2	16.3 ± 0.6	23.7 ± 0.7	692 ± 108	1.47
4-07/05/2009	10:56–12:36	2.03 ± 0.84	264 ± 14	36 ± 3	60 ± 5	22.8 ± 0.7	27.6 ± 0.6	784 ± 60	0.93

a Direction perpendicular to the windows for a *Poniente* (SW) wind is 208°.

**Table 2.** Ventilation volumetric flow rates through each vent opening calculated from Equation (13) with values of air velocities u^*^ corrected with wind speed: windward side *G_WS_*, leeward side *G_LS_* and windward roof *G_WR_*. Turbulent component of the volumetric flow rate *G*’ from Equation (14). Error in the calculation of ventilation flow rates *E_G_* and air exchange rate *R_M_*.

**Test**	***G_LS_* [m^3^ s^−1^]**	***G_WS_* [m^3^ s^−1^]**	***G_WR_* [m^3^ s^−1^]**	***G_M_* [m^3^ s^−1^]**	***E_G_* [%]**	***G’_LS_* [m^3^ s^−1^]**	***G’_WS_* [m^3^ s^−1^]**	***G’_WR_* [m^3^ s^−1^]**	***G’* [m^3^ s^−1^]**	***G’*/(*G_M_+G’*) [%]**	***R_M_* [h^−1^]**
1	−7.8	−5.3	14.1	13.6	7.5	4.1	4.0	8.2	16.3	54.5	18.2
2	−2.6	−2.7	4.0	4.6	−27.8	2.2	1.9	4.0	8.1	63.8	6.2
3	−3.3	−3.2	4.6	5.5	−33.6	2.6	2.3	4.0	8.9	61.8	7.4
4	0.6	−2.0	1.2	1.9	−11.4	1.7	1.2	2.6	5.5	74.3	2.6

**Table 3.** Values of outside radiation *R_g_*, the mean *Q* and turbulent *Q’* sensible heat fluxes by greenhouse surface for the windward (WS) and leeward side vents (LS), and the average mean and turbulent sensible heat exchanges (two bracketed terms).

**Test**	*Q̄_LS_***[W m^−2^]**	***Q’_LS_* [W m^−2^]**	*Q̄_WS_***[W m^−2^]**	*Q’_wS_***[W m^−2^]**	*Q̄_S_***[W m^−2^]**	*Q’_S_***[W m^−2^]**	$\left[\frac{\bar{u}\bar{\left(T-T_{o}\right)}}{u_{o}\Delta T_{\mathrm{io}}}\right]$	$\left[\frac{\bar{u\prime T\prime }}{u_{o}\Delta T_{\mathrm{io}}}\right]$
1	− 96.0 ^a^	−0.1	−63.0	−0.4	−158.9	−0.5	0.0513 (99.6%)	0.0002 (0.4%)
2	−23.3	−1.0	−22.9	0.1	−46.2	−0.9	0.0160 (98.2%)	0.0003 (1.8%)
3	−36.1	−0.9	−34.9	0.3	−70.9	−0.7	0.0276 (98.9%)	0.0003 (1.1%)
4	3.1	−4.2	−12.6	−1.1	−9.5	−5.3	0.0114 (63.7%)	0.0065 (36.3%)

a a negative sign indicates that sensible heat exits through the side vents.

**Table 4.** Mean value of the parameters that characterise the turbulent airflow close to the vents: *i*, turbulence intensity (longitudinal component, *x*; transversal component, *y*; vertical component, *z*); *k*, turbulence kinetic energy [m^2^ s^−2^]; *ɛ*, energy dissipation rate [m^2^ s^−3^].

**Test**	Vent *	***i_x_***	***i_y_***	***i_z_***	***i***	***k***	*ɛ*
**1**	**LS**	0.348 ± 0.061	0.274 ± 0.065	0.254 ± 0.044	0.297 ± 0.037	0.063 ± 0.023	0.081 ± 0.049
	**WS**	0.503 ± 0.109	0.337 ± 0.062	0.338 ± 0.075	0.374 ± 0.038	0.052 ± 0.017	0.108 ± 0.063
	**WR**	0.394 ± 0.019	0.576 ± 0.021	-	0.527 ± 0.024	0.303 ± 0.224	-
**2**	**LS**	0.451 ± 0.107	0.324 ± 0.055	0.331 ± 0.068	0.324 ± 0.036	0.020 ± 0.005	0.026 ± 0.010
	**WS**	0.402 ± 0.072	0.324 ± 0.051	0.402 ± 0.080	0.339 ± 0.038	0.017 ± 0.006	0.028 ± 0.015
	**WR**	0.577 ± 0.006	0.556 ± 0.050	-	0.526 ± 0.035	0.055 ± 0.028	-
**3**	**LS**	0.431 ± 0.120	0.311 ± 0.065	0.323 ± 0.082	0.321 ± 0.056	0.027 ± 0.009	0.040 ± 0.024
	**WS**	0.420 ± 0.061	0.326 ± 0.048	0.390 ± 0.072	0.343 ± 0.039	0.022 ± 0.007	0.035 ± 0.012
	**WR**	0.555 ± 0.032	0.562 ± 0.030	-	0.489 ± 0.030	0.056 ± 0.019	-
**4**	**LS**	0.621 ± 0.106	0.252 ± 0.067	0.415 ± 0.077	0.367 ± 0.049	0.007 ± 0.003	0.009 ± 0.008
	**WS**	0.400 ± 0.086	0.251 ± 0.040	0.358 ± 0.147	0.353 ± 0.064	0.005 ± 0.002	0.004 ± 0.003
	**WR**	0.777 ± 0.059	0.546 ± 0.059	-	0.553 ± 0.044	0.024 ± 0.003	-

* Vents: windward side *WS*; leeward side *LS*; windward roof *WR.*

**Table 5.** Slope of the energy spectrum *β* of the turbulent airflow close to the vents.

**Test**	Vent *	***β_x_***	***β_y_***	***β_z_***	***β***
**1**	**LS**	1.45 ± 0.15	1.26 ± 0.09	1.46 ± 0.13	1.49 ± 0.10
	**WS**	1.40 ± 0.13	1.17 ± 0.15	1.45 ± 0.18	1.42 ± 0.18
**2**	**LS**	1.62 ± 0.18	1.44 ± 0.14	1.74 ± 0.25	1.53 ± 0.16
	**WS**	1.40 ± 0.12	1.22 ± 0.15	1.68 ± 0.16	1.42 ± 0.16
**3**	**LS**	1.54 ± 0.14	1.40 ± 0.11	1.69 ± 0.21	1.46 ± 0.12
	**WS**	1.43 ± 0.09	1.21 ± 0.11	1.68 ± 0.18	1.43 ± 0.10
**4**	**LS**	2.14 ± 0.32	1.68 ± 0.33	1.96 ± 0.35	1.94 ± 0.35
	**WS**	1.78 ± 0.41	1.54 ± 0.40	1.96 ± 0.33	1.83 ± 0.28

* Vents: windward side *WS*; leeward side *LS*.

## Notation

*C_d_*: discharge coefficient
*C_p_*: air thermal capacity [J kg^−1^ K^−1^]
*C_w_*: wind effect coefficient
*E*(*f*): spectral density [m^2^ s^−1^]
*E_G_*: error in the calculation of volumetric flow rate [%]
*f*: frequency [Hz]
*E_Q_*: wind-related ventilation efficiency coefficient deduced from heat flux measurement
*G*: ventilation volumetric flow rate [m^3^ s^−1^]
*G′*: turbulent component of the ventilation volumetric flow rate [m^3^·s^−1^]
*HR*: relative humidity [%]
*i*: turbulence intensity
*k*: turbulence kinetic energy [m^2^ s^−2^]
*l*: two-dimensional horizontal resultant of air velocity in XY plane [m s^−1^]
*L_i_*: integral length scale [m]
*N*: number of measurement points in vents
*Q*: sensible energy flux exchanged between the greenhouse air and outside [W]
*R*: ventilation rate for greenhouse [h^−1^]
*R*(*t*): normalized autocorrelation function [m^2^ s^−2^]
*R_g_*: solar radiation [W m^−2^]
*S_A_*: greenhouse area [m^2^]
*S_V_*: surface area of the vent openings [m^2^]
*T*: temperature [°C]
*T′*: fluctuating air temperature [°C]
*t*: time [s]
*t_int_*: integral time scale [s]
*u*: air velocity [m s^−1^]
*ū_j_*: time-mean air velocity [m s^−1^]
*U′*: fluctuating component of air velocity [m s^−1^]
*u_o_*: wind speed [m s^−1^]
*X*(*f*): Fast Fourier Transform (FFT)
*X*(*t*): sample data
*X**(*f*): conjugate complex number of *X*

**Greek Letters**
Δ: difference
*β*: power spectrum exponent
*δ_t_*: time [s]
*ɛ*: turbulence energy dissipation rate [m^2^ s^−3^]
*θ*: wind direction [°]
*λ*: microscale [m]
*σ*: standard deviation

**Subscripts**
*i*: inside
*j*: measurement point
*L*: leeward
*M*: average value
*o*: outside
*R*: roof vent
*S*: side vent
*W*: windward
*x*: longitudinal component
*y*: transversal component
*z*: vertical component
